# Myogenic progenitor cell transplantation for muscle regeneration following hindlimb ischemia and reperfusion

**DOI:** 10.1186/s13287-021-02208-w

**Published:** 2021-02-24

**Authors:** Franka Messner, Marco Thurner, Jule Müller, Michael Blumer, Julia Hofmann, Rainer Marksteiner, Sebastien Couillard-Despres, Jakob Troppmair, Dietmar Öfner, Stefan Schneeberger, Theresa Hautz

**Affiliations:** 1 Daniel Swarovski Research Laboratory (DSL), Department of Visceral, Transplant and Thoracic Surgery (VTT), Center of Operative Medicine, Medical University of Innsbruck (MUI), Innrain 66, 6020 Innsbruck, Austria; 2 Innovacell Biotechnologie AG, Innsbruck, Austria; 3 Department of Anatomy, Histology and Embryology, Division of Clinical and Functional Anatomy, Medical University of Innsbruck, Innsbruck, Austria; 4 Institute of Experimental Neuroregeneration, Spinal Cord Injury and Tissue Regeneration, Center Salzburg (SCI-TReCS), Paracelsus Medical University, Salzburg, Austria; 5 Austrian Cluster for Tissue Regeneration, Vienna, Austria; 6 Department of Visceral, Transplant and Thoracic Surgery, Center of Operative Medicine, Medical University of Innsbruck, Anichstrasse 35, 6020 Innsbruck, Austria

**Keywords:** Satellite cells, Myogenic progenitor cells, Stem cell, Ischemia-reperfusion injury, Transplantation, Muscle regeneration

## Abstract

**Background:**

Muscle is severely affected by ischemia/reperfusion injury (IRI). Quiescent satellite cells differentiating into myogenic progenitor cells (MPC) possess a remarkable regenerative potential. We herein established a model of local application of MPC in murine hindlimb ischemia/reperfusion to study cell engraftment and differentiation required for muscle regeneration.

**Methods:**

A clamping model of murine (C57b/6 J) hindlimb ischemia was established to induce IRI in skeletal muscle. After 2 h (h) warm ischemic time (WIT) and reperfusion, reporter protein expressing MPC (TdTomato or Luci-GFP, 1 × 10^6^ cells) obtained from isolated satellite cells were injected intramuscularly. Surface marker expression and differentiation potential of MPC were analyzed in vitro by flow cytometry and differentiation assay. In vivo bioluminescence imaging and histopathologic evaluation of biopsies were performed to quantify cell fate, engraftment and regeneration.

**Results:**

2h WIT induced severe IRI on muscle, and muscle fiber regeneration as per histopathology within 14 days after injury. Bioluminescence in vivo imaging demonstrated reporter protein signals of MPC in 2h WIT animals and controls over the study period (75 days). Bioluminescence signals were detected at the injection site and increased over time. TdTomato expressing MPC and myofibers were visible in host tissue on postoperative days 2 and 14, respectively, suggesting that injected MPC differentiated into muscle fibers. Higher reporter protein signals were found after 2h WIT compared to controls without ischemia, indicative for enhanced growth and/or engraftment of MPC injected into IRI-affected muscle antagonizing muscle damage caused by IRI.

**Conclusion:**

WIT-induced IRI in muscle requests increased numbers of injected MPC to engraft and persist, suggesting a possible rational for cell therapy to antagonize IRI. Further investigations are needed to evaluate the regenerative capacity and therapeutic advantage of MPC in the setting of ischemic limb injury.

**Supplementary Information:**

The online version contains supplementary material available at 10.1186/s13287-021-02208-w.

## Background

Ischemia is characterized by a restriction of blood supply. Following to subsequent reperfusion, an ischemia-reperfusion injury (IRI) with inflammation and damage to organs and tissues is induced [1]. During this phase, excessive generation of reactive oxygen species (ROS) prompt tissue inflammation and mitochondrial dysfunction, which may result in cell death and negatively impacts organ and tissue function [2, 3].

Prolonged ischemia times remain a major obstacle in salvage of extremities in the context of acute and critical limb ischemia (ALI/CLI), and vascularized composite allotransplantation (VCA). Muscle tissue has been identified to be most susceptive to IRI [4, 5]. In ischemic muscle biopsies, a variable degree of inflammatory infiltration, tissue damage, and elevated expression levels of proinflammatory cytokines and perivascular inflammatory infiltrates have been observed [5–7].

Stem cells (cell therapy) represent an emerging novel therapeutic option for the treatment of IRI. Multipotent mesenchymal stromal cells (MSC) isolated from bone marrow and adipose tissue have the ability to differentiate into multiple cell lineages and hence to compensate tissue damage [8]. MSC have the capacity to differentiate into skeletal myogenic cells in vitro and in vivo [9]. However, ex vivo proliferation—inevitable to produce sufficient cell numbers for therapeutic application—results in the loss of the skeletal myogenic differentiation potential [10]. Another cell type useful for skeletal muscle regeneration are myogenic stem cells, which possess a remarkable regenerative potential for skeletal muscles [11, 12]. These cells reside between the basal lamina and the sarcolemma of myofibers. Activation of myogenic stem cells leads to differentiation into proliferating myogenic progenitor cells (MPC), which in turn fuse to new myofibers to support regeneration of damaged muscle tissue [11]. The importance of these cells in muscle regeneration was demonstrated in PAX7KO mice [13, 14], where a reduced regenerative potential was induced by ablation of MPC. Furthermore, intramuscular transplantation of in vitro expanded MPC was successfully used for treatment of skeletal muscle deficiency related fecal incontinence in the clinics [15–17]. This makes MPC an excellent candidate for cell therapy.

We established a murine hindlimb ischemia/reperfusion injury model for the assessment of engraftment and regenerative potential of in vitro expanded and intramuscularly injected MPC.

## Methods

### Experimental animals and study groups

Eight to 12-week-old male SHO-Prkdc^scid^Hr^hr^ mice and C57BL/6 J (H-2^Kb^) weighing between 25 and 30 g were used. Animals were purchased from Charles River (Germany) and housed under standardized conditions with unrestricted access to water and food. All experiments were approved by the Austrian Federal Ministry of Science and Research (66.011/0191-WF/V/3b/2016) and the Health Department of the State Government of Salzburg, Austria (20901-TVG/96/7-2014). To establish cell injection parameters (dose determination and number of needles), SHO-Prkdc^scid^Hr^hr^ mice were used, whereas C57BL/6 J mice were utilized to establish the ischemic muscle injury model. A detailed overview on experimental groups is provided in Table 1.

**Table 1:** Overview experimental groups

Experimental groups					
Subgroups	WIT	Cell amount	Cell type	Endpoint	Number
**Baseline testing:** MPC implantation and tracking was established (SHO-Prkdc^scid^Hr^hr^ mice)					
	None	0.5 mio. cells	Luci_GFP	POD 35	*n* = 2
	None	1 mio. cells	Luci_GFP	POD 35	*n* = 2
	None	1 mio. cells—1 needle	Luci_GFP	POD 71	*n* = 4
	None	1 mio. cells—4 needles	Luci_GFP	POD 71	*n* = 4
	None	1 mio. cells—4 needles	TdTomato	POD 70	*n* = 2
**Group A:** Characterization of warm ischemic injury in a model of murine limb ischemia (C57BL/6 mice)					
**A1**	30 min	None	–	POD 3, 7, 14	*n* = 3
**A2**	1 h	None	–	POD 3, 7, 14	*n* = 3
**A3**	2 h	None	–	POD 3, 7, 14	*n* = 3
**A4**	3 h	None	–	POD 3, 7, 14	*n* = 4
**Group B:** Myogenic progenitor cells and ischemia/reperfusion injury (C57BL/6 mice)					
**B1**	2 h	None	Puffer	POD 2	*n* = 8
**B2**	2 h	1 mio. cells—4 needles	TdTomato	POD 2	*n* = 5
**Group C:** Myogenic progenitor cells and muscle regeneration (C57BL/6 mice)					
**C1**	2 h	None	Puffer	POD 14	*n* = 8
**C2**	2 h	1 mio. cells—4 needles	TdTomato	POD 14	*n* = 5
**Group D:** Ischemic injury and cell engraftment (C57BL/6 mice)					
**D1**	2 h	1 mio. cells—4 needles	Luci_GFP	POD 75	*n* = 7
**D2**	None	1 mio. cells—4 needles	Luci_GFP	POD 75	*n* = 8

*WIT* warm ischemic time, *POD* postoperative day

### Surgical procedure

#### Clamping model of murine hind limb ischemia

Animals were sedated with isoflurane (Baxter GmbH, Austria; 3% for induction, 1.5–2% for maintenance) and analgesia was performed with intraperitoneally administered buprenorphine (0.1 mg/kg; Temgesic®, Reckitt Benckiser Healthcare Ltd., UK). After skin disinfection, a circumferential incision was made in the groin. The epigastric vessels were cauterized and transected and the femoral vessels exposed. First, the femoral artery and then the femoral vein were dissected and side branches were transected after cauterization. Under preservation of the femoral and sciatic nerve branches, the ventral and dorsal muscle groups were transected at the level of the mid-thigh to prevent collateral perfusion of the hind limb. The femoral artery and vein were clamped using two vessel clamps (Supplementary Fig. 1). The animal was kept under anesthesia for the duration of warm ischemic time (WIT, ranging from 30 min (min) to 3h in a pilot study). Reperfusion was achieved by the release of vessel clamps. If applicable, MPC (detailed description see below) or sham (5 μL FluoSpheres® polystyrene beads, [15 μm, yellow-green or scarlet; Thermo-Fisher Scientific, USA] and 25 μL1XPBS) injections (groups B–D) were carried out right after reperfusion in the *tibialis anterior* muscle (Supplementary Fig. 2 A). The individual muscle groups of the thigh were approximated with 6-0 Vicryl (Ethicon Inc., USA) and skin closure was performed using 6-0 Prolene (Ethicon Inc., USA). Animals were monitored on a heating pad until recovery from surgery.

#### Surgical exposure for MPC injection without ischemia

Animals were sedated with isoflurane (Baxter GmbH, Austria; 3% for induction, 1.5–2% for maintenance), and analgesia was performed with intraperitoneally administered buprenorphine (0.1 mg/kg; Temgesic®, Reckitt Benckiser Healthcare Ltd., UK). After disinfection, a longitudinal incision was made along the ventral aspect of the tibia. The *tibialis anterior* muscle was then exposed and MPC were injected i.m. (Supplementary Fig. 2 B). Skin closure was performed with 6-0 Prolene and animals were monitored on a heating pad until recovery from surgery.

### MPC isolation and cultivation

Cells were obtained from skeletal muscle biopsies of adult B6-albino.Gt(ROSA)26Sortm4(ACTB-tdTomato,-EGFP)Luo/J/PMU or adult B6-albino.FVB-TG(CAG-Luc-GFP)L2G85Chco/J/PMU following cervical dislocation. Skeletal muscle tissue was obtained from *longissimus dorsi*, *gastrocnemius*, and *tibialis anterior muscles*, transferred into a sterile petri dish and covered with 1X PBS. Cells were isolated as described before [18]. In short, muscles were cut into 2–4 mm-sized segments and enzymatically digested using the skeletal muscle dissociation kit (MiltenyiBiotec, Germany) following the manufacturer’s instructions. In order to separate MPC from non-myogenic cells (NMC), a satellite cell isolation kit (Miltenyi Biotec, Germany) was used according to the manufacturer’s instructions. Cultivation, cryopreservation, cell count, and harvest were performed as described before [19]. Briefly, cells were cultivated in growth medium consisting of DMEM/Ham’s F12 medium (Gibco®, Thermo-Fisher, USA) supplemented with 20% FCS (Thermo-Fisher, USA), bFGF (Cellgenix, Germany), and Gentamicin (Sandoz, Germany). Cells were cultivated at 37 °C and 5% CO_2_. Sub-cultivation was performed by detachment of cells with Trypsin/EDTA in 1x PBS (Sigma-Aldrich, USA). Cryopreservation of cells was realized by controlled rate freezing (− 1°/min) of cells suspended in cryopreservation medium (Ringer’s lactate, 5% DMSO, 10% serum albumin) to liquid nitrogen. MPC of Luciferase mice were used for quantification of the engraftment, distribution, and persistence of cells in host muscle following intramuscular implantation. MPC of TdTomato mice were used for studying the engraftment of cells in host tissue on a histological level.

### Immunocytochemistry

Immunofluorescent staining was performed as described earlier [19]. For fluorescent immunolabeling of desmin, cells were consecutively incubated with rabbit anti-desmin (Thermo Scientific, USA) antibodies, and donkey anti-rabbit Alexa488 conjugated antibodies (Thermo Scientific, USA). Counterstaining of nuclei was performed by incubating the cells with Hoechst33342 (Sigma-Aldrich Co. LLC, USA) diluted to a final concentration of 2 μg/mL in PBS. Fluorescence was visualized under a standard fluorescence microscope Nikon Eclipse TE 2000-U microscope (Nikon Corporation, Japan).

Immunocytochemical staining was performed in order to visualize desmin protein expression in isolated cells. For this, 200,000 cells were seeded in growth medium on a gelatin-coated well of a 24-well plate and incubated for 24 h. Next, cells were washed by aspirating medium and adding 1× PBS. After aspiration of PBS, 500 μL of 4% paraformaldehyde was added to the cells and incubated at room temperature (RT) for 10 min for fixation. After washing the cells three times with each 500 μL PBST (0.1% Triton-X-100 in 1x PBS), cells were covered with Ultravision Hydrogen Peroxide Block (Thermo Fisher, USA) and incubated for 5 min at RT. After three additional washing steps with PBST, cells were covered with rabbit anti-desmin antibodies (Thermo-Fisher, USA) diluted 1:100 in blocking medium (0.1% Triton-X-100, 3% normal goat serum in 1X PBS) and incubated at 37 °C for 90 min. Cells were washed again with PBST and covered with ready-to-use horseradish peroxides conjugated goat anti-rabbit secondary antibodies (Thermo Fisher, USA) and incubated for 60 min at 37 °C. Afterwards, cells were washed again with PBST, followed by incubation with Lab Vision™ Ready-To-Use AEC Substrate System (Thermo Fisher Scientific) for 10 min at RT. The reaction was stopped by washing the cells with 1X PBS three times. Counterstaining of nuclei was performed in addition by covering cells with Harris Hematoxylin (Sigma-Aldrich, USA) for 10 min and washed with 1X PBS to remove residual staining solution. Cells were visualized on a Nikon Eclipse TE2000-U inverted Microscope (Nikon Corporation, Tokyo, Japan).

### Syto24 live-cell staining of nuclei

500,000 living cells were pelleted by centrifugation at 400×*g* for 7 min followed by resuspending the cells in 500 μL Ringer’s Lactate solution (Fresenius-Kabi, Germany) containing 0.5 μM Syto24 nuclear dye (Thermo-Fisher, USA). Cells were then incubated for 90 min at 4 °C followed by addition of 10 mL Ringer’s lactate solution. Next, cells were centrifuged, supernatant discarded, and cell pellet resuspended in 10 mL growth medium. After another centrifugation, cell pellet was resuspended in growth medium to achieve 125,000 cells per mL.

### Fusion competence analysis

Cells were seeded in growth medium on wells of a 24-well plate coated with 0.1% gelatin in 0.9% NaCl (CellGenix, Germany). Coating was performed by adding 500 μL of coating solution to each well and incubation of the plate for 30 min at RT. Afterwards, the coating solution was aspirated and 125.000 cells in 1 mL were directly seeded and allowed to attach for 24 to 48 h. Afterwards, differentiation was induced by aspirating the growth medium and adding 1 mL skeletal muscle cell differentiation medium (PromoCell, Germany), supplemented with 2% of skeletal muscle cell differentiation medium Supplement Mix (PromoCell, Germany) and 0.05% gentamicin solution (8 mg/mL, Sandoz, Austria). Finally, cells were incubated at 37 °C, 5% CO_2_ for 4–7 days without further medium change.

### Acetylcholinesterase activity analysis

Acetylcholinesterase (AchE) activity measurement was performed as described before [19]. In short, medium was carefully removed from cells grown on a 24-well plate followed by the immediate addition of 300 μL 0.5 mM DTNB solution (prepared in phosphate buffer, pH 7.2 with 0.1% triton X-100). After 2 min of incubation at RT in the dark, 50 μL of 5.76 mM ATI (prepared in distilled water) was added. The reagent mixture was incubated for 60 min at 30 °C in the dark followed by the OD measurement at 412 mM on an Anthos Zenyth 340rt microplate reader (Biochrom Ltd., UK). AchE activity (mUrel) was normalized per gram protein of lysed cells.

### Flow cytometry

To determine surface marker expression of MPC, flow cytometry was performed on a Guava easyCyte 6HT 2 L flow cytometer (Merck Millipore, Germany). The BD Lyoplate™ Mouse Cell Surface Marker Screening Panel containing purified monoclonal antibodies specific for inter alia CD9 and CD98 cell surface marker proteins (BD biosciences, USA) was employed according to manufacturer’s instructions. Cell events were acquired with Guava InCyte™ v.2.3 software. Histograms and dot- plots were generated with a minimum of 5000 events at a sample flow rate of 1.8 μL/mL. Positive staining was obtained by comparison with isotype control set as at least 95% negative or comparison to control (negative) cells.

### MPC injections following ischemia/reperfusion

Cryopreserved MPC were freshly thawed, washed once with 1X PBS, and centrifuged at 400×*g* for 10 min followed by resuspension in 1X PBS to reach a final concentration of 40 × 10^6^ cells/mL. Twenty-five microliters of the MPC suspension (containing 1 × 10^6^ cells) were mixed on a Parafilm with 5 μL FluoSpheres® polystyrene beads (15 μm, yellow-green or scarlet, Thermo-Fisher Scientific, USA), in order to track MPC within the *tibialis anterior* muscle during follow-up (Supplementary Fig. 2C). MPC were injected intramuscularly using a custom-made injector (Innerbichler GmbH, Austria) containing 4 needles (30G) mounted on a 1 mL syringe (Braun, Germany).

### In vivo bioluminescence imaging and quantification

Luciferase reporter protein intensity of MPC isolated from Luciferase mice was visualized and quantified by in vivo imaging using IVIS Spectrum system (PerkinElmer, MA, USA) to analyze cell engraftment, persistence, and migration. Mice were injected with the substrate of luciferase, D-Luciferin (Sigma-Aldrich Co. LLC, USA) dissolved in 1X PBS at a concentration of 30 mg/mL. 150 mg D-Luciferin per kilogram (KG) bodyweight was applied intraperitoneally. Mice were then anesthetized with isoflurane (Baxter GmbH, Austria; 3% for induction) and placed within the bioimager under continuous isoflurane inhalation (1.5–2% for maintenance). Using the Living Image® software (PerkinElmer, USA) bioluminescence signals were detected once per minute for a total of 30 min and overlaid with photographs of the mice for signal localization. To quantify the signal, the total bioluminescence signal (total flux per specified field of ROI) within the expected body area of every measurement was background corrected and plotted against the measurement time.

### Histology

Muscle tissue was fixed in 4% paraformaldehyde and paraffin-embedded. 6 μm paraffin sections were made and stained with hematoxylin and eosin (H&E) and Mason Goldner staining as per standard procedures. Sections were examined with a Zeiss Axioplan 2 (Zeiss, Germany) microscope and photographed using a Zeiss AxioCam HR and AxioVision 4.1. software running on a Pentium 4 (Intel Inc. Santa Cruz, USA) with Windows XP (Microsoft Inc., Redmond, USA). Histologic evaluation and regeneration scoring (Table 2) were performed by an experienced histologist blinded for experimental groups belonging.

**Table 2:** Histopathologic muscle regeneration scale

*Muscle regeneration scale for histopathology*	
0	No signs of muscle regeneration
1	Mild signs of muscle regeneration
2	Moderate signs of muscle regeneration
3	Severe signs of muscle regeneration
Histological signs of muscle regeneration include: • Cytoplasmic basophilia (early after damage) • Presence of myoblast/myotubes • Rows of internal, centrally located nuclei • Vesiculated nuclei with prominent nucleoli	

### Immunohistochemistry

Paraffin-embedded histological sections were de-paraffinized by consecutively submerging and incubating the slides for each 5 min twice in xylene, 100% EtOH, 90% EtOH, 70% EtOH, and finally tap water. In order to retrieve potentially blocked antigens in paraffinized sections, antigen retrieval was performed following de-paraffinization. Sections were submerged in a boiling antigen retrieval buffer (0.01 M sodium citrate, 0.05% Tween, pH 6.0 in dH_2_O) and incubated for 30 min, followed by rinsing the slides in PBST (0.1% Tween in 1x PBS).

For immunohistochemistry, sections were permeabilized and unspecific binding sites were blocked by incubation with blocking medium (0.1% TritonX-100), 3% goat-Serum in 1x PBS) for 1 h. Afterwards, slides were incubated overnight with rabbit anti-desmin (Thermo-Fisher, USA) and/or goat-anti TdTomato (SICGEN, Portugal) primary antibodies diluted 1:100 in blocking medium at 4 °C. After rinsing and washing with PBST, slides were incubated for 2 h at 4 °C with secondary donkey anti-rabbit Alexa488 (Thermo-Fisher, USA) and/or donkey anti-goat Alexa568 (Thermo-Fisher, USA) antibodies each diluted 1:200 in blocking medium. Following rinsing and washing with PBST, slides were incubated for 20 min with Hoechst or DAPI diluted to 2 μg/ml in PBS and embedded in Entellan® (Merck, Germany).

### Statistics

Depending on distribution, Student’s *t* test and analysis of variance (ANOVA) or Mann-Whitney *U* test were used for inter-group comparison. A two-sided *p* value of < 0.05 was considered statistically significant. GraphPad Prism 6 (GraphPad Software Inc., USA) was used for all analyses. Results are expressed as median and range or mean and standard deviation (SD) for continuous variables and counts and percentages for discrete variables. Kaplan-Meier survival curves and log-rank test were used to determine differences in animal survival between groups. Bioluminescence signal was calculated by determining the area under the curve over time.

## Results

### MPC characterization

**Fig. 1:**
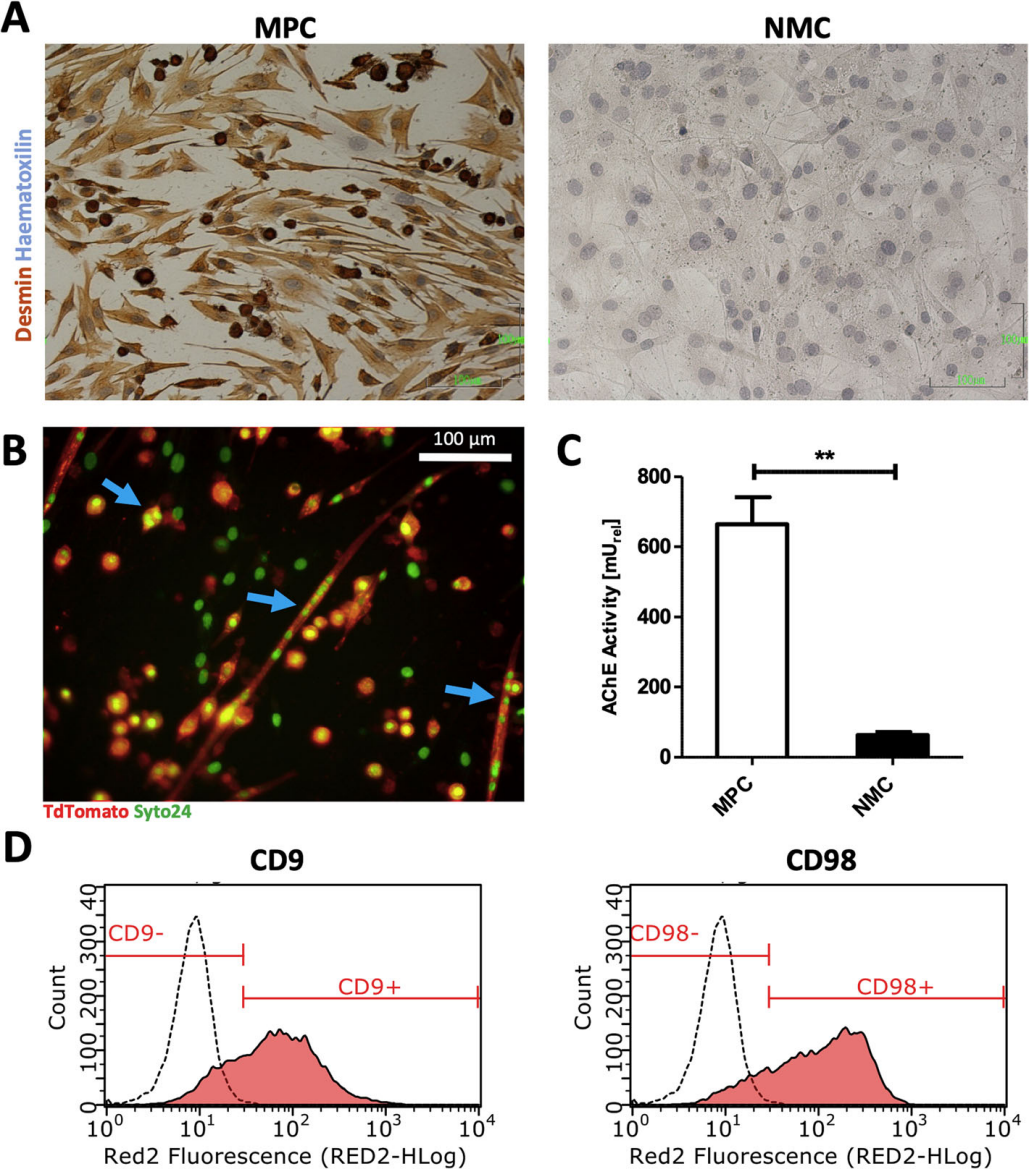
Cell characterization. **a** Immunocytochemical staining of MPC and NMC demonstrating positive desmin expression in MPC (red color) and negative desmin expression in NMC. Nuclear counterstaining performed by hematoxylin. **b** Live-cell imaging of Syto24 stained TdTomato MPCs, cultivated in skeletal muscle differentiation medium for 4 days (blue arrows: multinucleated myotubes). **c** Quantification of AChE activity per 2 × 10^5^ cells in either MPC or NMC both cultivated in skeletal muscle differentiation medium. **d** Flow cytometric analysis of CD9 and CD98 surface marker in MPC from at least two different mice demonstrated as histogram (red). Isotype control stained cells (white histograms) used to determine threshold for positivity. AChE, acetylcholinesterase; MPC, myogenic progenitor cell; NMC, non-myogenic cells

MPC isolated from skeletal muscles of TdTomato and Luciferase positive mice were analyzed for their myogenic identity and skeletal muscle differentiation potential in vitro. Desmin, a general myogenic cell marker and intermediate type filament necessary for muscle contraction [20], stained positive in 94.67 ± 9.24% (mean ± SD) of MPC compared to non-myogenic cells (NMC) (Fig. 1a). Furthermore, MPC cultivated under differentiating conditions was found to be fusion-competent and formed multinucleated myotubes (Fig. 1b). Moreover, MPC demonstrated significantly higher AChE activity compared to NMC (Fig. 1c), suggesting high skeletal myogenic differentiation potential of MPC. CD9, a tetraspanin surface marker required for normal fusion of myotubes and muscle regeneration [21], as well as CD98, a surface marker identifying activated muscle stem cells [22], were found highly positive at a mean ± SD of 79.75 ± 8.45 (CD9) % and 86.71 ± 7.54 (CD98) % in MPC, respectively (Fig. 1d).

### Cell implantation: dose definition and cell distribution

**Fig. 2:**
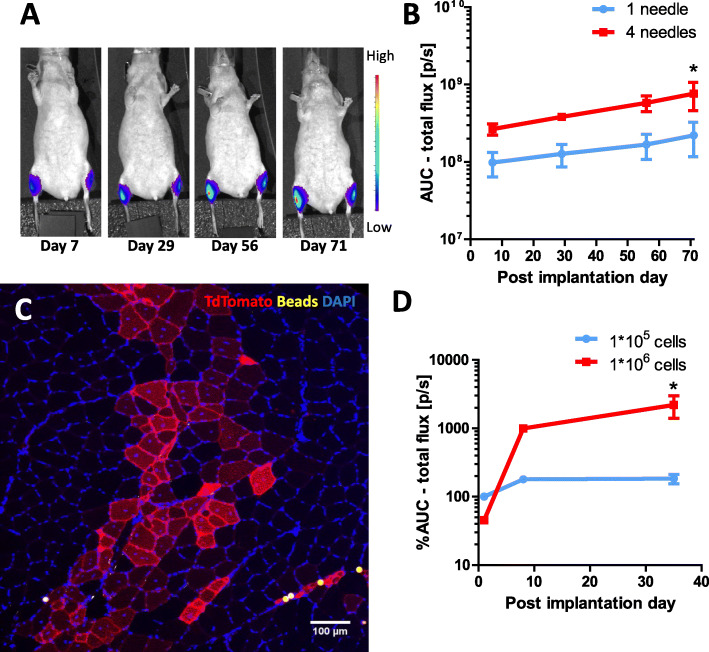
MPC implantation model. **a** Bioluminescence intensity (BLI) over time of a representative mouse injected with fixed number and volume (1 × 10E6 cells in 25 μL+ 5 μL beads) of Luciferase-MPC and fluorescent beads into the tibialis anterior muscles either by a single-needle (left hindlimb) or 4-needle applicator (right hindlimb). BLI images representing 30 min measurements of luciferase specific photon emission set to a standardized scale (rainbow). **b** Luciferase signal quantification (area under the curve; AUC) of 4 mice receiving 1 × 10E6 Luciferase-MPC either by 1-needle injection (left *M. tibialis* anterior) or 4 needle injection (right *M. tibialis* anterior). Luciferase signals quantified by background corrected area under the curve of 30 min (1 measurement per minute) analysis on 7, 29, 56, and 71 days post cell implantation. Data presented as mean and SD. **c** Section of TdTomato MPC and fluorescent beads-injected mouse stained with anti-TdTomato and anti-Laminin antibodies as well as DAPI (Scale bar = 200 μm). **d** Luciferase signal quantification by cell dose of two mice either receiving 1 × 10E5 or 1 × 106E6 cells injected in the right and left m. gastrocnemius, respectively on days 1, 7, and 35 post implantations demonstrated as % AUC of low cell dose at day 1. AUC, area under the curve; BLI, bioluminescence; DAPI, 4′,6-diamidin-2-phenylindol; MPC, myogenic progenitor cell; SD, standard deviation

In order to study the fate of MPC following intramuscular injection, an in vivo model of MPC implantation and tracking was established. MPC from Luciferase or TdTomato mice were implanted into the *tibialis anterior* or *gastrocnemius* muscle of immunodeficient SHO-Prkdc^scid^Hr^hr^ mice. It has previously been shown that intramuscularly injected myogenic cells do not migrate well [23]. Thus, to distributed MPC over the whole muscle, a multiple needle applicator with 4 needles (each 30G) placed at a distance of 1 mm from each other was designed (Supplementary Fig. 3). Signals of Luciferase MPC injected by single or multi-needles were visible at the site of injection over the whole study course of 75 days (Fig. 2a, b), suggesting long-term engraftment of cells in both cases. Significantly higher luciferase signals were found when cells were injected by a 4-needle multi-needle applicator compared to those injected by a single-needle applicator, suggesting that a 4-needle distribution is favorable to reach high cell engraftment in the subsequent ischemia approach (Fig. 2b). Immunohistological analysis of muscle specimen injected with TdTomato cells revealed TdTomato positive myofibers on POD 70 (Fig. 2c), suggesting fusion of MPC with existing myofibers and/or formation of new myofibers. Successful engraftment of injected cells is necessary for myofiber formation and thus hypothesized to be essential for regenerative effects of MPC. Therefore, an effort was made to increase MPC engraftment. Optimal cell dose per needle for an injection depth of 0.25 mm was calculated to be 2.5 × 10^5^ cells per needle according to Skuk et al. (1 × 10^6^ total for a 4-needle applicator) [24]. Comparison of luciferase signals emitted by either 1 × 10^6^ or 1 × 10^5^ cells injected per muscle over time revealed significantly lower signals in muscles injected with fewer cells (Fig. 2d), thus confirming dose-signal relation and cell dose definition. Again, luciferase signals significantly increased over time until POD 75, thus suggesting ongoing proliferation of injected MPC (Fig. 2d).

### Hindlimb ischemia/reperfusion model

**Fig. 3:**
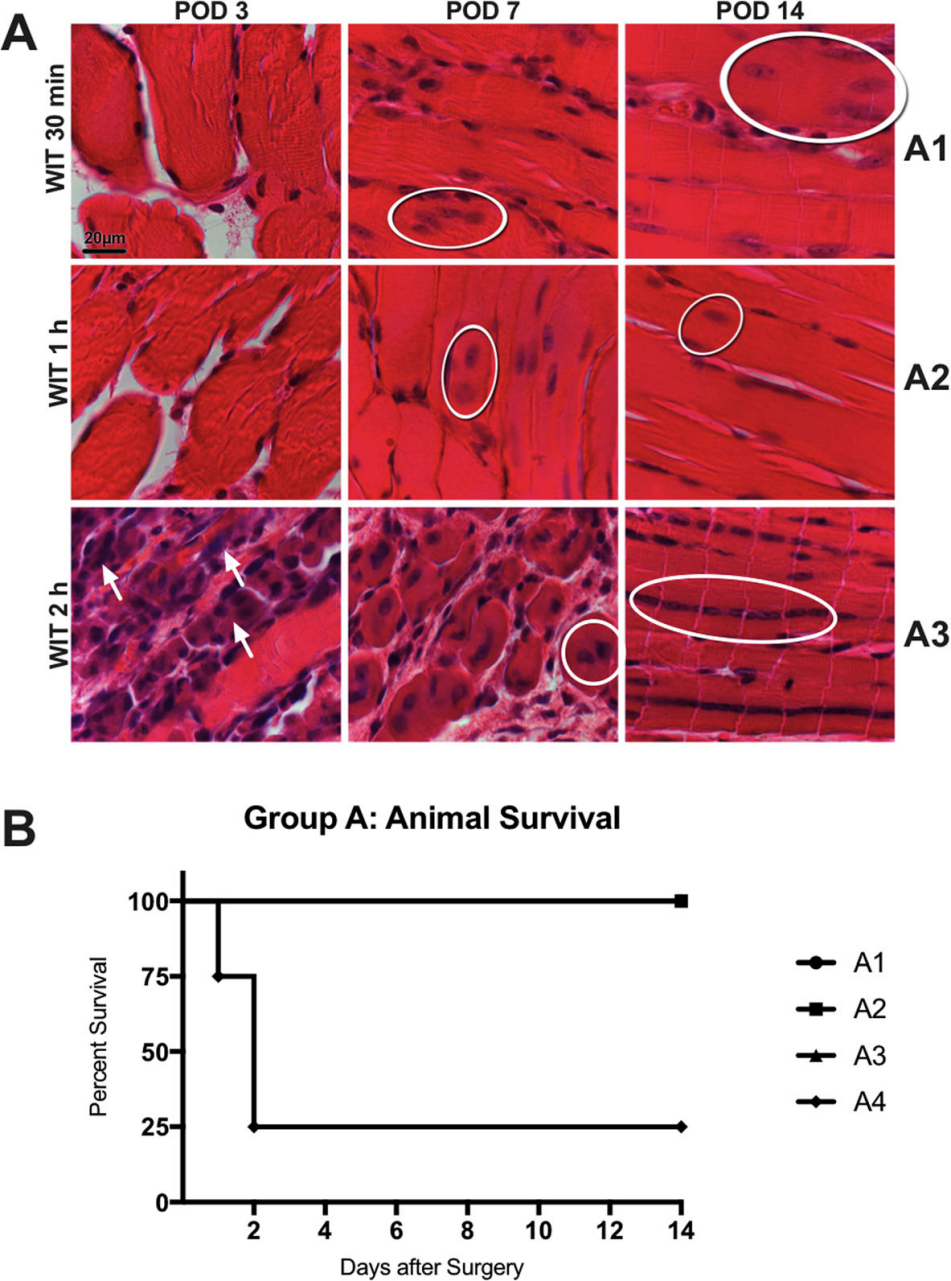
Characterization of and animal survival after ischemic injury. **a** Muscle tissue injury after 30 min, 1 h and 2 h of WIT in a murine hindlimb clamp model assessed on POD 3, 7, and 14. Histological signs of regeneration indicated by centrally located cell nuclei were observed on POD 7 and 14 (white circle) in all investigated groups. Prolonged WIT lead to more pronounced ischemic muscle injury. **b** Animal survival was 100% for animals subjected to a WIT up to 2 h but significantly worsened thereafter. Only 25% of the animals in the 3h WIT group reached the endpoint on POD 14. POD, postoperative day; WIT, warm ischemic time

To establish a murine model of muscle damage due to warm ischemia and reperfusion in extremities, WIT ranging from 30 min to 3h was tested with the goal to induce muscle damage without major muscle necrosis. Short WIT (group A1, 30 min WIT) did not lead to macroscopic and only minimal histopathologic changes (Fig. 3a), including few internal nuclei with prominent nucleoli. Macroscopic signs of IRI including progressive swelling and erythema of the ischemically injured leg were observed in animals subjected to prolonged (≥ 1 h) WIT (data not shown). Histopathologic evaluation of muscle biopsies in groups A2 (1 h WIT) and A3 (2h WIT) demonstrated mild and moderate to severe leukocyte infiltrations on POD 3 and 7, respectively (Fig. 3a). In addition, signs of muscle regeneration were seen in biopsies by POD 14 (Fig. 3a). Especially muscle tissue in group A3 displayed characteristic cytoplasmatic basophilia on POD 3 and internal rows of vesiculated nuclei with prominent nucleoli on POD 14 (Fig. 3a, bottom row). While animal survival was excellent in groups A1 to A3, a sharp decline in animal survival to 25% was observed after increasing WIT to 3h in group A4 (Fig. 3b). As animals in group A3 displayed signs of moderate to severe ischemic injury and excellent postoperative survival rates, 2h of WIT were identified as most suitable to investigate the regenerative properties of myogenic progenitor cells.

### MPC engraftment and persistence following IRI in vivo

**Fig. 4:**
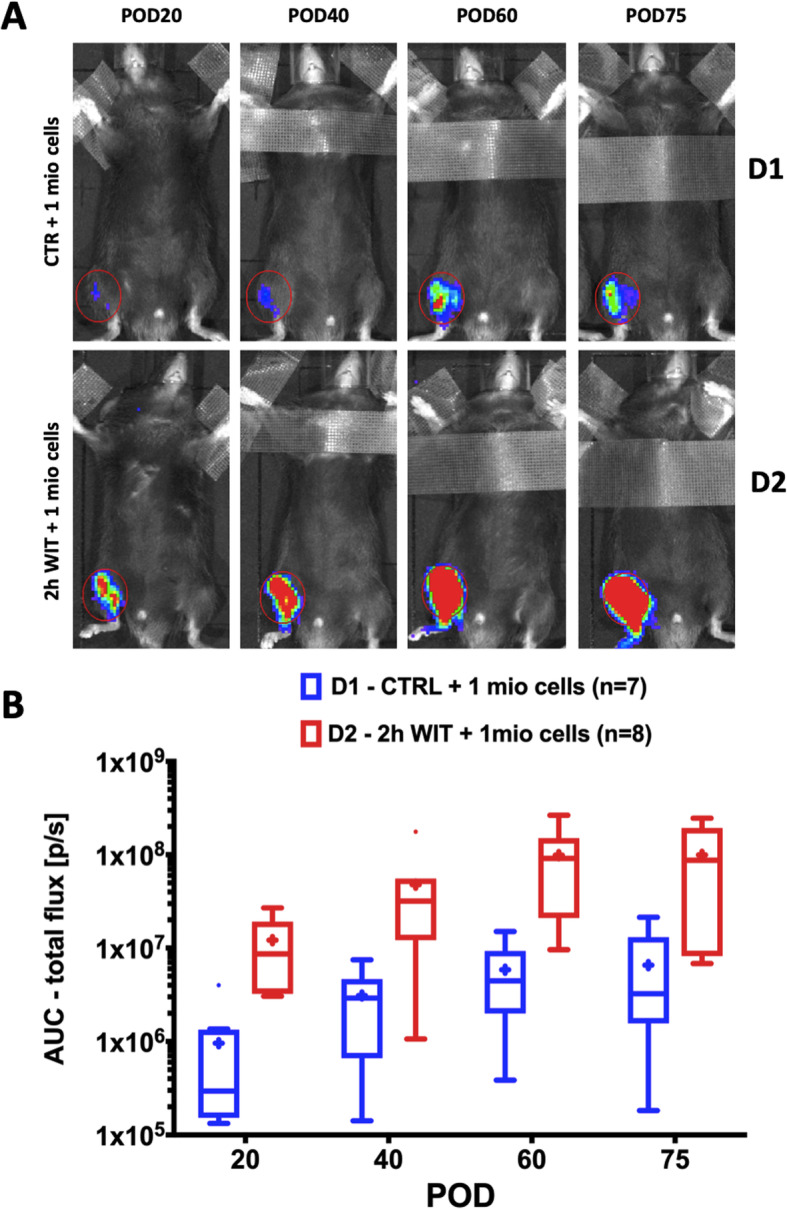
Engraftment and persistence of MPC following ischemia. **a** Representative bioluminescence images of mice, which had their right hindlimb either subjected to 2h WIT (D2, *n* = 8) or not (D1, *n* = 7) followed by injection of 1 mio luciferase reporter expressing MPC into the *tibialis anterior* muscle. Depicted images represent 1 of 30 images taken on POD 20, 40, 60, and 75 show bioluminescence signals as heatmap (red = high, blue = low signal). Red circles depict area for quantification of signals. **b** Luciferase signal quantification (area under the curve; AUC) of mice receiving intramuscular injection of luciferase MPC, following 2h warm ischemia (WIT2H, D2) or no ischemia (CTR, D1). Luciferase signals quantified by background corrected area under the curve of 30 min (1 measurement per minute) analysis on day 20, 40, 60, and 75 post cell implantation (POD). Data presented as Tukey’s boxplots (mean shown as “+”). MPC, myogenic progenitor cell; POD, postoperative day; WIT, warm ischemic time

In order to study the engraftment and persistence of implanted MPC following IRI, luciferase reporter expressing MPC were injected into the *tibialis anterior* muscle of C57BL/6 mice without WIT (group D1) or after 2h WIT (group D2). Bioluminescence of injected cells was visible throughout the entire study period (POD 75) in all mice of groups D1 and D2 (Fig. 4a). The signal persisted at the area of injection without further distribution. Quantification of the bioluminescence signals demonstrated increasing signals over time in both study groups (*p* < 0.001) and consistently higher signals in the 2h WIT (D2) animals (Fig. 4b) (*p* = 0.0155). These findings suggest that MPC engraft and persist at the injection site, with a higher number of cells present after IRI.

### Interplay of MPC engraftment, tissue damage, and regeneration following IRI in vivo

**Fig. 5:**
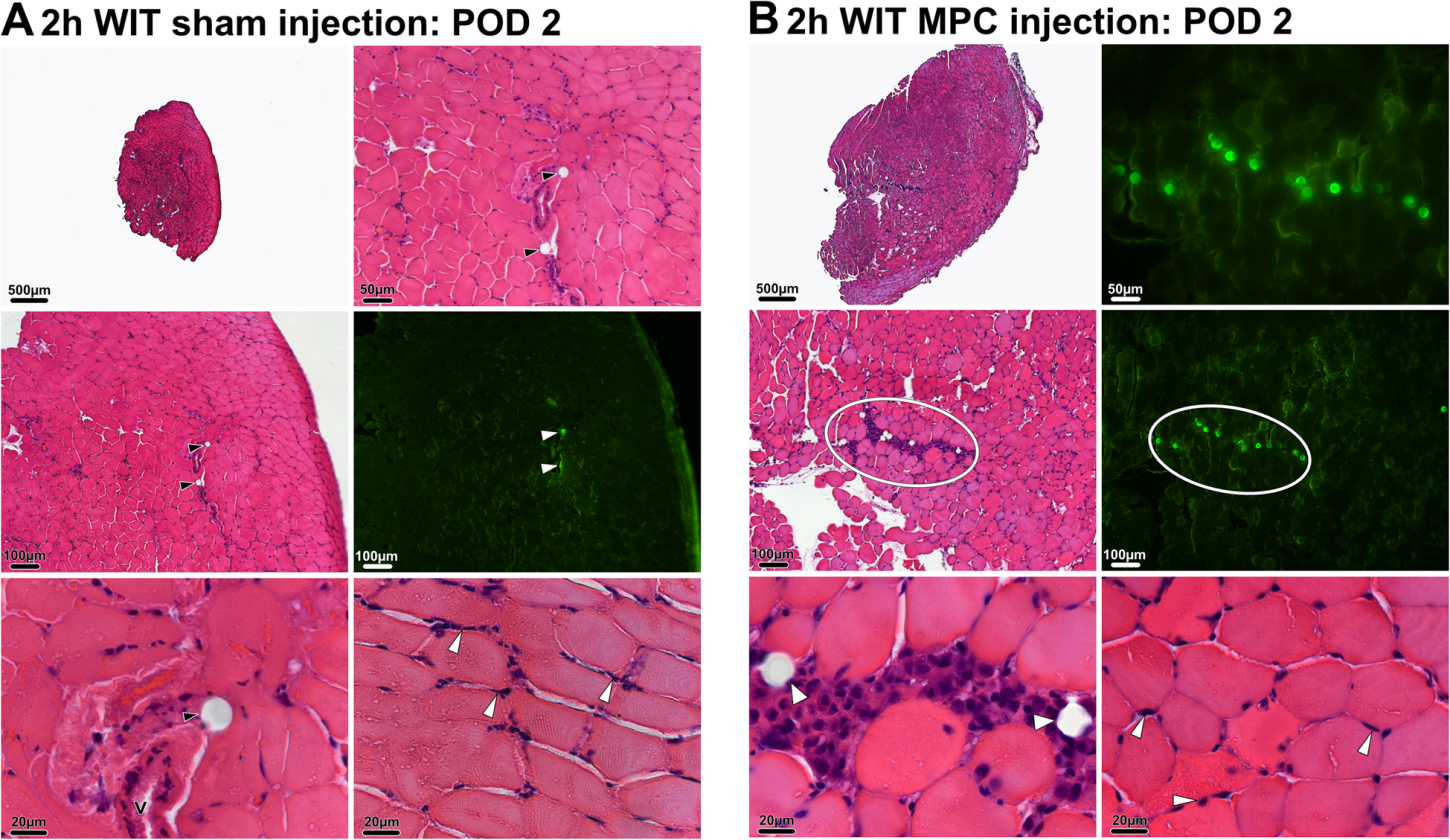
Histological analysis POD 2. Both sham injected (group B1) and MPC injected (group B2) animals displayed moderate signs of ischemic injury after 2h WIT assessed by H&E staining. After sham (**a**) and cell (**b**) injections, FluoSpheres® polystyrene beads (black and white arrow heads, white circles) were detectable in the *tibialis anterior* muscle. Their presence was confirmed by fluorescence microscopy. In contrast to sham-injected animals, a dense cellular aggregate surrounding the co-injected beads was observed (**b**, left column). H&E, hematoxylin and eosin; MPC, myogenic progenitor cell; POD, postoperative day; V, vessel, WIT, warm ischemic time

**Fig. 6:**
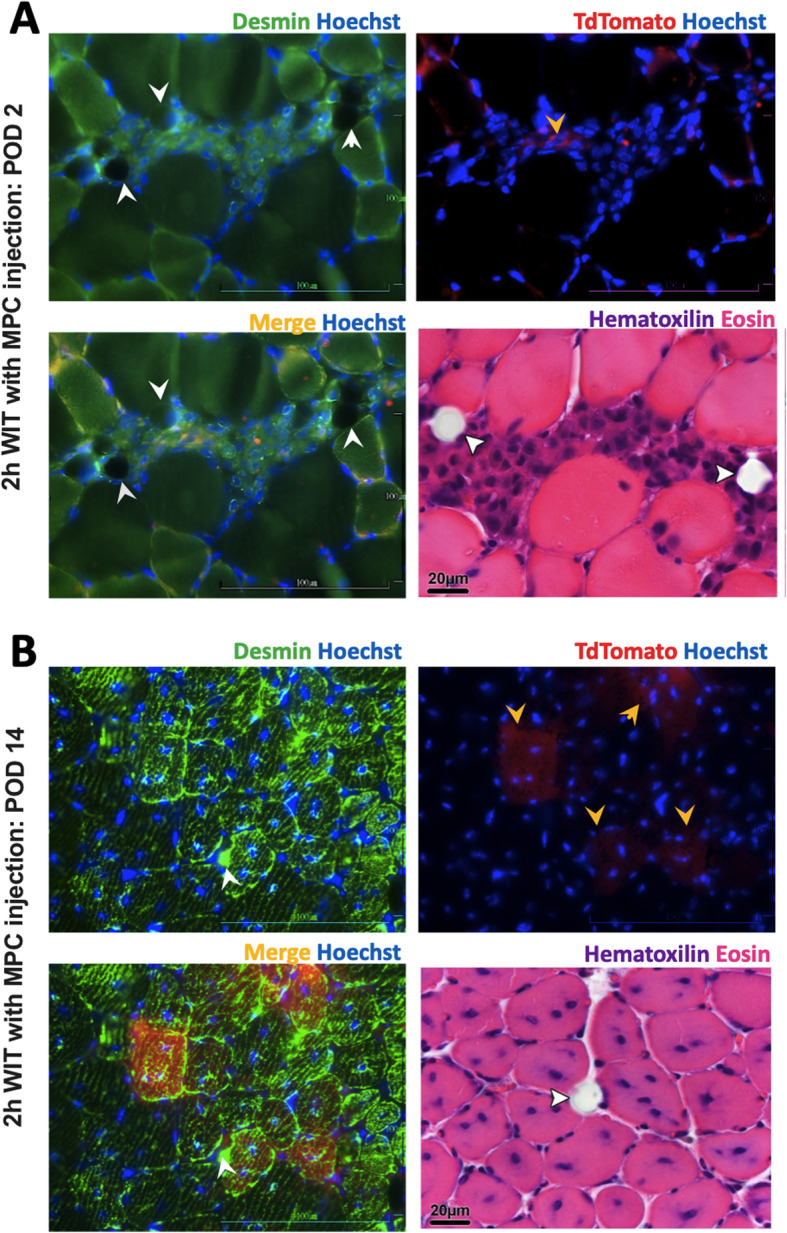
Regenerative mode of action of MPC. *Tibialis anterior* sections of mice obtained 2 (**a**) or 14 (**b**) days after 2h WIT and intramuscular injection of MPC. Sections were stained for desmin and TdTomato protein expression and Hoechst3344 or H&E. White arrows indicate fluorescent beads or reminiscent holes of beads. Yellow arrows indicate TdTomato positive cells (**a**) or myofibers (**b**). H&E, hematoxylin and eosin; MPC, myogenic progenitor cell; WIT, warm ischemic time

Biopsies of the *tibialis anterior* muscle from animals subjected to 2h WIT taken on POD 2 (group B) displayed moderate to severe leukocyte infiltration without signs of major muscle necrosis. No difference in the extent of leukocyte infiltration or muscle cell damage was observed between animals receiving sham (B1, Fig. 5a) or MPC injections (B2, Fig. 5b). In contrast to animals in group B1, accumulating cell infiltrates were seen around the co-injected beads in all animals of group B2 (Fig. 5b, bottom row). TdTomato and desmin+ cells were detected at these sites (Fig. 6a), suggesting that injected cells remained at the injection site co-located with fluorescent beads.

**Fig. 7:**
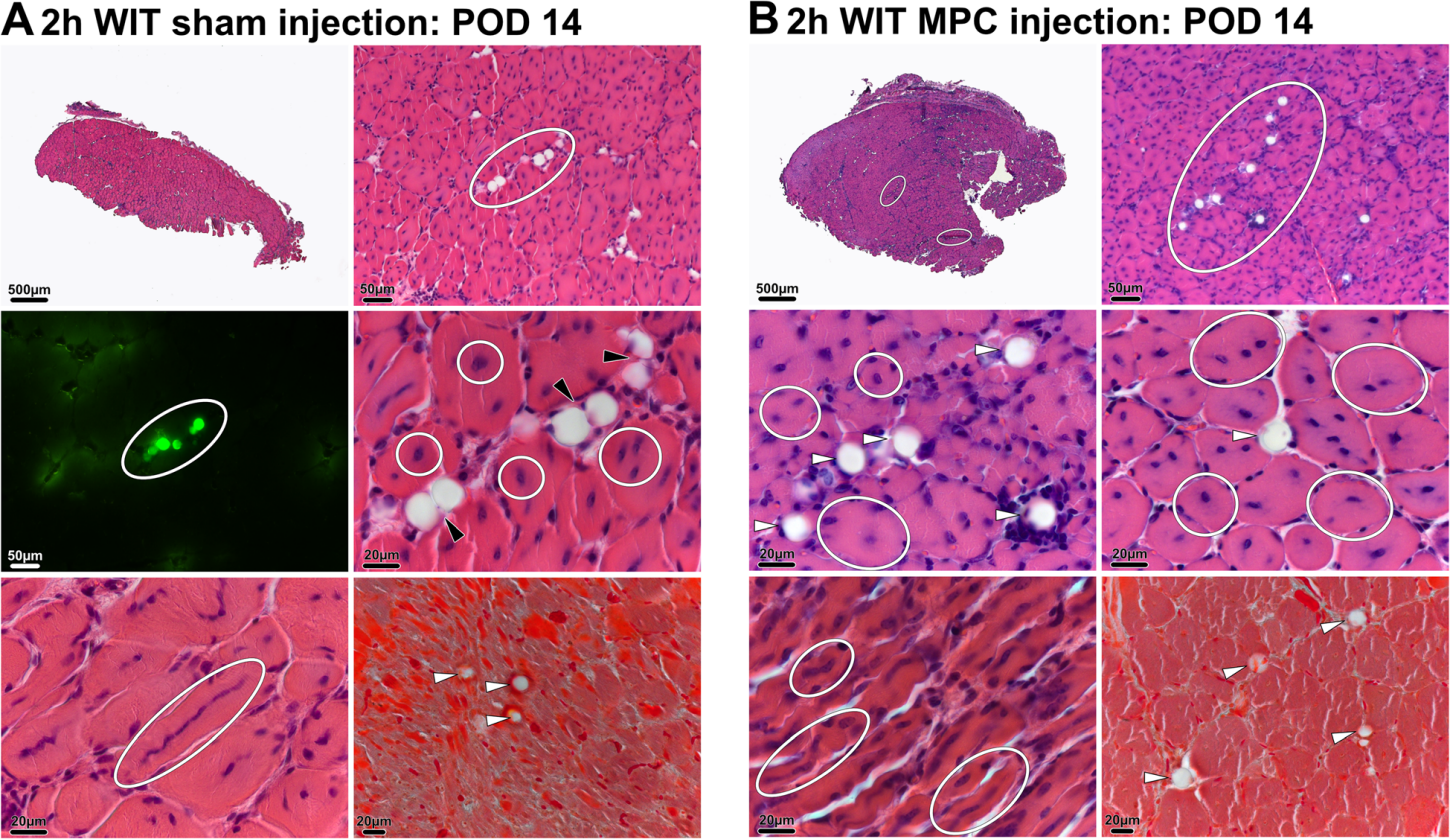
Histological analysis POD 14. Fourteen days after hindlimb ischemia, animals with sham (**a**) and intramuscular injection of MPC (**b**) demonstrated distinct signs of regeneration. In animals with cell injections H&E staining showed cellular aggregates surrounding the co-injected polystyrene beads (white and black arrows and white circles). In comparison to the observed cell piles on POD 2, however, they tend to be less dense. Masson-Goldner staining (bottom rows right picture) revealed that neither cell nor sham injections lead to scar formation or tissue fibrosis. H&E, hematoxylin and eosin; MPC, myogenic progenitor cell; POD, postoperative day

**Fig. 8:**
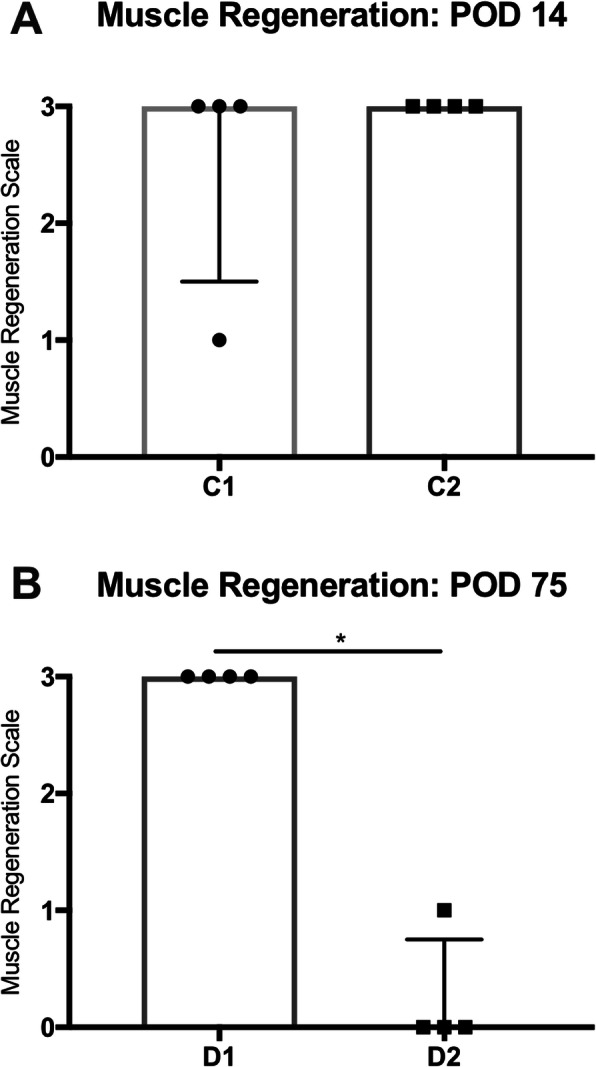
Assessment of muscle regeneration after warm ischemic injury. In order to quantify muscle generation, histological slides were analyzed and graded by a blinded histologist according to a 4-tier scale (0 = no signs of regeneration, 1 = mild signs of regeneration, 2 = moderate signs of regeneration, 3 = severe signs of regeneration). **a** Fourteen days after ischemic injury with 2h of WIT, both sham (C1) and cell injected (C2) animals displayed similar signs of regeneration. **b** In animals with ischemic injury (D1) and MPC-injection, a significantly higher muscle regeneration score was seen on POD 75 compared to animals without ischemic injury and MPC-injection. For intergroup comparison, the Mann-Whitney test was used. *p < 0.05; MPC, myogenic progenitor cell; POD, postoperative day; WIT, warm ischemic time

On POD 14, pronounced muscle regeneration as evident from the presence of myotubes with multiple internal vesiculated nuclei and prominent nucleoli, known to occur following fusion of single nucleated muscle progenitor cells with each other [25], was observed in all groups challenged by WIT (Fig. 7a and b, black circles). Animals with sham injection (C1) and with MPC injection (C2) both displayed a median muscle regeneration score (Table 2) of 3 (range, C1: 1–3; C2: 3–3; *p* > 0.9) (Fig. 8a). On POD 14, only few MPC were left aggregated around beads in C2 mice (Fig. 7b, middle row). As assessed through fluorescence imaging, the TdTomato signal, originally expressed by injected MPC, was located in newly formed (central nuclei containing) desmin expressing myofibers, indicating that injected MPC have contributed to myofiber regeneration (Fig. 6b). Accordingly, a low degree of fibrosis and tissue damage was observed in muscle biopsies of both groups (Fig. 7a and b, bottom row). In animals with ischemic injury (D1) and MPC-injection, a significantly higher muscle regeneration score was seen on POD 75 compared to animals without ischemic injury and MPC-injection (Fig. 8b).

**Fig. 9:**
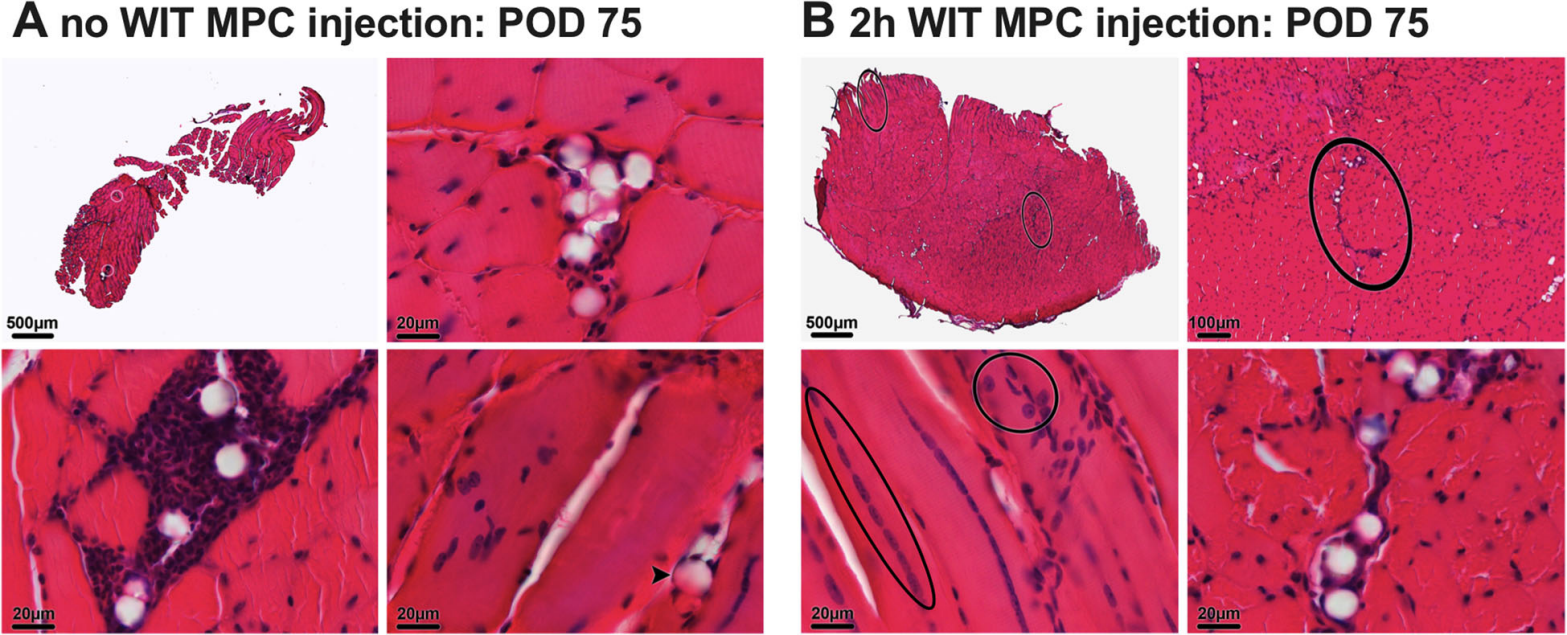
Histological analysis POD 75. H&E staining of the *tibialis anterior* muscle 75 days after intramuscular injection of MPC in **a** uninjured muscle and **b** muscle subjected to 2h WIT. While no cellular aggregates were found in animals after ischemic injury (**b**), dense cell piles were seen in uninjured muscle samples (**a**, bottom left). In animals subjected to 2h WIT centrally located nuclei and thus signs of regeneration (**b**, bottom left, black circles) were still present on POD 75. H&E, hematoxylin and eosin; MPC, myogenic progenitor cell; WIT, warm ischemic time

Seventy-five days after MPC injection, only very few cells were still present in close proximity to the co-injected beads in ischemically injured animals of group D1 (Fig. 9b). This was in stark contrast to histopathologic results from animals in group D2 (MPC injection in sham operated animals, no WIT), where large aggregates of cells were still visible around the co-injected beads (Fig. 9a), suggesting that IRI increased the cellular turnover in infiltrates at the injection site. In group D1, a high degree of muscle regeneration was still seen on POD 75 reflecting in a median regeneration score of 3 (range, 3–3). In contrast, animals in group D2 displayed little to no muscle tissue regeneration and thus a median score of 0 (range, 0–1; *p* = 0.029; Fig. 9b), suggesting that IRI led to an increase in muscle regeneration. Similarly to earlier time-points, no relevant amounts of fibrosis and scaring were seen 75 days after MPC injection.

## Discussion

In this study, we investigated the therapeutic potential of MPC, which are known for their beneficial properties regarding isolation and in vitro cultivation [12]. We successfully isolated murine MPC with a high skeletal muscle potency indicated by a high desmin expression as well as AChE activity, which is required for muscle regeneration [19, 26]. In line with previous studies in different animal models [23, 24, 27, 28], we were able to demonstrate not only that intramuscularly injected MPC contribute to myofiber formation. Our study reveals that MPC engraftment and persistence was feasible in immunodeficient mice, as well as in syngeneic immunocompetent host mice. In addition, warm ischemic injury further significantly contributed to increased cell engraftment in mice. Our data are in line with findings of Zhang et al. who applied MPC intramuscularly to a Duchenne muscular dystrophic mouse model and reported the generation of new muscle fibers; however, this study was lacking a non-diseased wild-type control group [29].

There is evidence that the success of MSC engraftment highly depends on the route of administration. Despite reports suggesting that MSC successfully home to injured muscle after intravenous injection [30], the favored route of administration in clinical trials was intramuscular injection [31]. In a previous study, Braid et al. compared intravenous with intramuscular administration of MSC in a mouse model. Cell tracking showed prolonged cell survival for the intramuscular administration route [32]. Thus, we chose to utilize the intramuscular injection route in our study. Due to limited mobility of injected MPC, we observed that an even distribution upon injection within the muscle tissue was crucial for optimal cell distribution. Cell injections with a single-needle applicator lead to MPC engraftment; however, the luciferase signal intensity was significantly increased when using a needle applicator with 4 needles in our mice.

Similar to preconditioning regimens in bone marrow or stem cell transplantation, the generation of space or a certain degree of tissue damage for the injected cells seems to be essential for their integration and fusion in skeletal muscle. Incitti et al. showed an increase of myogenic potential once cells reached their cell niche [33]. Furthermore, homing of the administered stem cells at the site of ischemic injury was found in various small animal models of ischemic diseases [34–36]. These observations are in line with our results. By injecting cells in ischemically injured as well as in naïve, non-injured muscle tissue and subsequent in vivo tracking of these cells, we were able to show that the signal intensity was significantly increased in animals with ischemic injury. This demonstrates better cell engraftment and differentiation in injured muscle tissue also in our animals.

By visualizing the tdTomato signal in cell aggregates surrounding co-injected beads on POD 2, we could confirm that those cells indeed represented injected MPC and not cellular infiltrate. On POD 14, the tdTomato signal was detected in regenerating muscle fibers in close proximity to co-injected beads suggesting that injected MPC contributed to muscle regeneration in this model. At the study endpoint on POD 75, myotubes with internal rows of multiple nuclei and prominent nucleoli were still present in ischemically injured and treated animals. This finding was in contrast to non-injured MPC-treated animals where cell aggregates of MPC were observed 75 days after injection and a generally lower bioluminescence signal was detected hinting at higher level of cellular turnover and repair in WIT-treated animals. Histologic analysis and immunohistochemistry of muscle biopsies suggested that injected MPC contributed to the generation of new or restoration of existing muscle fibers upon ischemia-induced damage.

In the proposed study, male mice were used for all experiments. As sex-related differences in muscle recovery after IRI have been described before [37], the results obtained in our study might not extend to female mice and further studies evaluating this gender-specific aspect are warranted. We would further like to point out that MPC therapy protocols have already been introduced in the clinics in various settings of muscle regeneration, each with promising results [15, 16, 29, 38, 39]. Skuk et al. [40] described a case of healthy muscle-precursor cell transplantation in a patient with Duchenne muscular dystrophy (DMD). Long-term expression of donor-derived dystrophin in injected muscle tissue and lack of side effects were reported in this case. Another study [17] explored the use of autologous skeletal muscle-derived cells for treatment of fecal incontinence. A total of 39 patients received injection of 79.4 × 10^6^ cells in the external anal sphincter, and 12 months after treatment, the number of weekly incontinence episodes, Fecal Incontinence Quality of Life, and patient condition had improved significantly.

Moreover, the therapeutic use of a combination of MPC and MSC seems to hold great potential and exert a beneficial effect in terms of muscle regeneration in human. Klimczak et al. [39] performed co-transplantation of MPC and bone marrow derived MSC in three patients suffering from DMD. While no adverse effects were observed after intramuscular administration, the authors demonstrated that treated muscle displayed donor cell protein expression, suggesting cell engraftment. But also clinical parameters also improved after treatment, such as an increase in motor unit parameters, and decreased levels of creatine kinase and pro-inflammatory cytokines. Future studies will have to focus on differential, convergent and synergistic effects of MPC and MSC in human muscle regeneration to foster the development of optimal treatment protocols in terms of safety and efficacy.

## Conclusions

In summary, our data demonstrate that 2h of WIT-induced IRI in murine hindlimb skeletal muscle enable increased numbers of injected MPC to engraft and persist, suggesting a possible rational for cell therapy to antagonize IRI. Despite first promising results in this preclinical small animal model, further investigations are still warranted to evaluate the regenerative capacity and therapeutic advantage of MPC in the setting of clinical ischemic limb injury as well as the underlying mechanisms contributing to enhanced muscle regeneration.

## Supplementary Information

**Additional file 1.**

## Data Availability

The datasets used and/or analyzed during the current study are available from the corresponding author on reasonable request.

## Abbreviations

ALI: Acute limb ischemia
ATI: Acetylthiocholine iodide
bFGF: Basic Fibroblast Growth Factor
BM-MSC: Bone marrow mesenchymal stem cells
CLI: Critical acute limb ischemia
dH_2_O: Deionized water
DTNB: 5,5′-Dithiobis (2-nitrobenzoic acid)
DMD: Duchenne muscular dystrophy
EtOH: Ethanol
IRI: Ischemia/reperfusion injury
MPC: Myogenic progenitor cells
MSC: Mesenchymal stem cells
NMC: Non-myogenic cells
OD: Optical density
PBS: Phosphate buffered saline
PBST: PBS with Tween
POD: Postoperative day
ROI: Region of interest
ROS: Reactive oxygen species
RT: Room temperature
VCA: Vascularized composite allotransplantation
WIT: Warm ischemic time
