# Supplementary material for: Myogenic progenitor cell transplantation for muscle regeneration following hindlimb ischemia and reperfusion
Source: Stem Cell Res Ther. 2021 Feb 24;12:146. doi: 10.1186/s13287-021-02208-w (PMC7905585; doi:10.1186/s13287-021-02208-w)
Supplement: Supplementary file 1 — Additional file 1. [file 13287_2021_2208_MOESM1_ESM.docx]

**Supplementary Material**

**Supplementary Figure 1 – Surgical model** Exposure and vessel clamping during the induction of warm ischemic injury in the mouse hind limb. Applying the (A) arterial and (B) venous vessel clamps after transection of the (C) ventral and dorsal muscle groups of the thigh while preserving all (D) major nerve branches allows for reliable induction of warm ischemic injury.


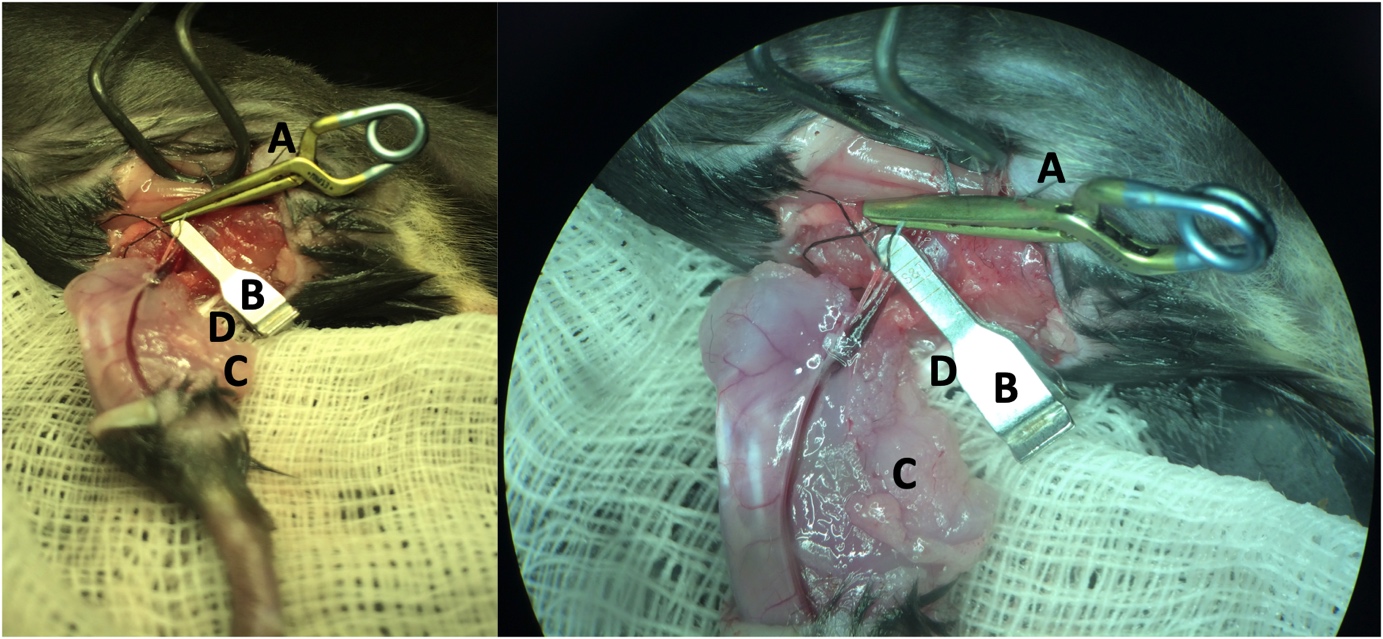


**Supplementary Figure 2 – Cell injection (**A) Injections of MPCs were performed in the *tibialis anterior* muscle after release of vascular clamps and successful reperfusion using a custom-made injector containing 4 needles (30G) mounted on a 1 ml syringe. (B) Sham injections were performed after exposure of the *tibialis anterior* muscle. (C) Co-injected FluoSpheres® polystyrene beads (15 µm, yellow-green) were visible macroscopically after injection in the *tibialis anterior* muscle. G, gauge; MPC, myogenic progenitor cell.


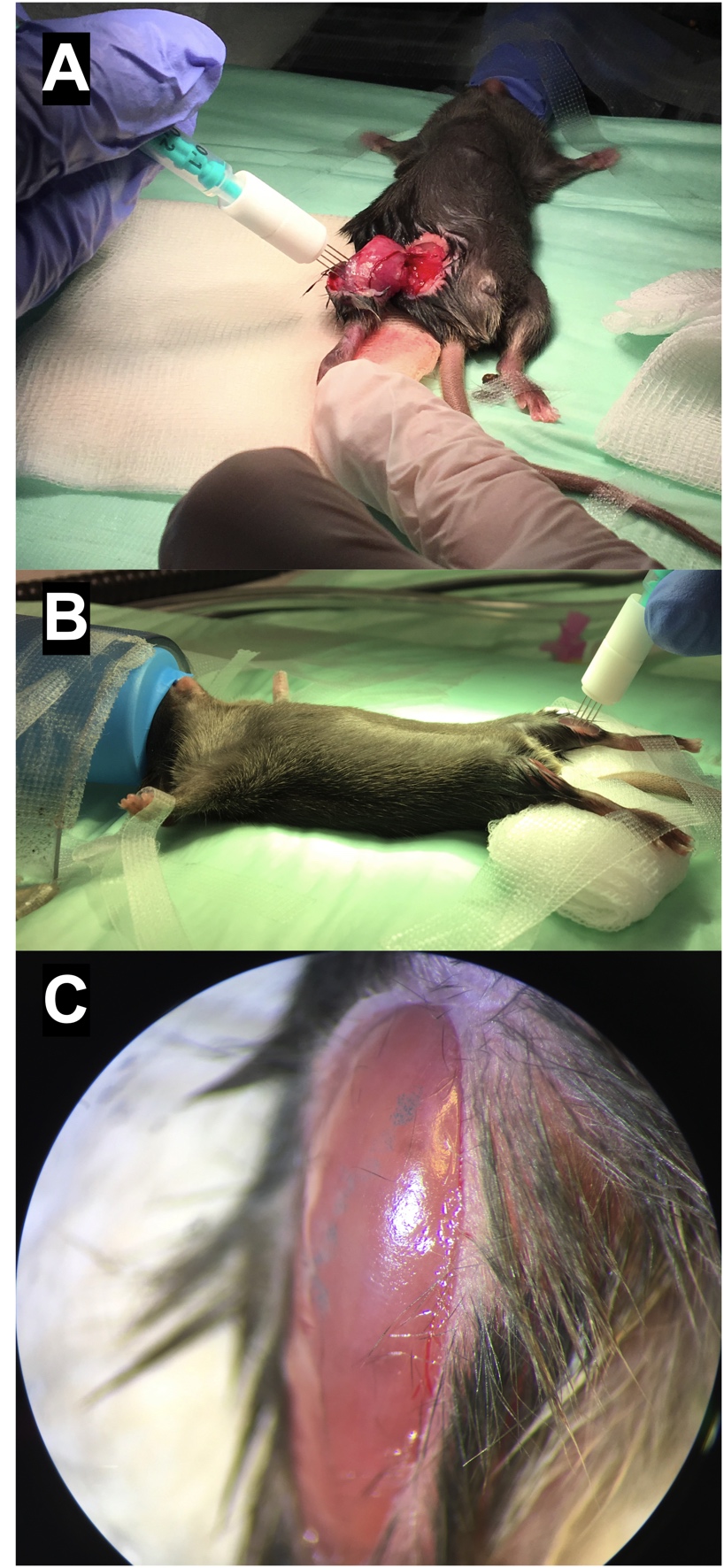


**Supplementary Figure 3 – Customized injectors** In order to assess the best mode of injection, different custom-made injectors were tested. While a standard 30 G injection needle consists of one needle (A), custom-made injectors containing 2 (B), 4 (C), and 8 (D) needles (30 G) were tested. The injector containing four needles (C) was most effective in cell aspiration, injection, and distribution of cells. With the injector containing 8 needles, aspiration of the 30 µl injection volume was not possible without aspiration of air. G, gauge.

**
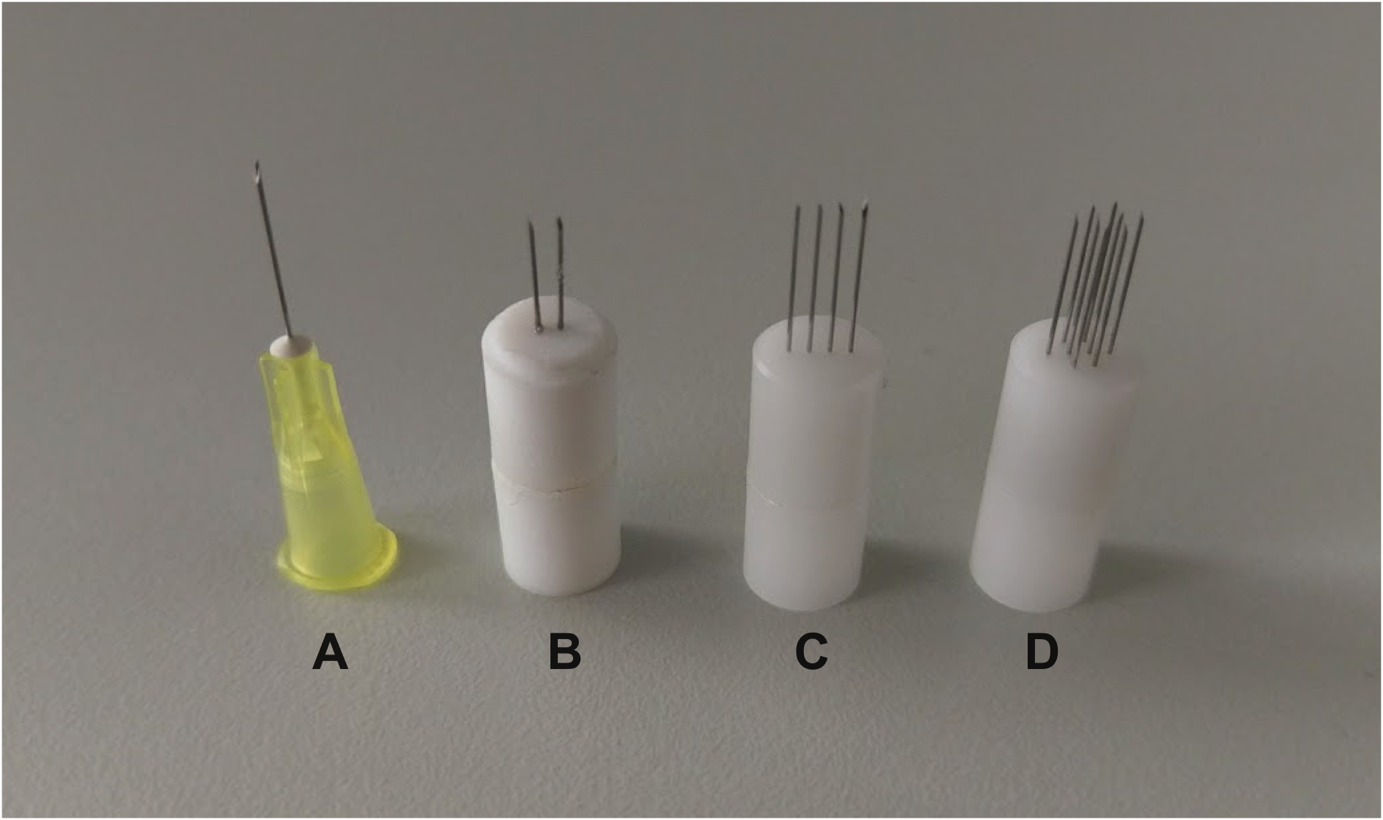
**
